# First Investigation of the Microbiology of the Deepest Layer of Ocean Crust

**DOI:** 10.1371/journal.pone.0015399

**Published:** 2010-11-05

**Authors:** Olivia U. Mason, Tatsunori Nakagawa, Martin Rosner, Joy D. Van Nostrand, Jizhong Zhou, Akihiko Maruyama, Martin R. Fisk, Stephen J. Giovannoni

**Affiliations:** 1 College of Oceanic and Atmospheric Sciences, Oregon State University, Corvallis, Oregon, United States of America; 2 Department of Earth Sciences, Tohoku University, Miyagi, Japan; 3 Petrology of the Oceanic Crust, Universität Bremen, Bremen, Germany; 4 Department of Botany and Microbiology, Institute for Environmental Genomics, University of Oklahoma, Norman, Oklahoma, United States of America; 5 Research Institute of Biological Resources, National Institute of Advanced Industrial Science and Technology, Ibaraki, Japan; 6 Department of Microbiology, Oregon State University, Corvallis, Oregon, United States of America; Argonne National Laboratory, United States of America

## Abstract

The gabbroic layer comprises the majority of ocean crust. Opportunities to sample this expansive crustal environment are rare because of the technological demands of deep ocean drilling; thus, gabbroic microbial communities have not yet been studied. During the Integrated Ocean Drilling Program Expeditions 304 and 305, igneous rock samples were collected from 0.45-1391.01 meters below seafloor at Hole 1309D, located on the Atlantis Massif (30 °N, 42 °W). Microbial diversity in the rocks was analyzed by denaturing gradient gel electrophoresis and sequencing (Expedition 304), and terminal restriction fragment length polymorphism, cloning and sequencing, and functional gene microarray analysis (Expedition 305). The gabbroic microbial community was relatively depauperate, consisting of a low diversity of proteobacterial lineages closely related to Bacteria from hydrocarbon-dominated environments and to known hydrocarbon degraders, and there was little evidence of Archaea. Functional gene diversity in the gabbroic samples was analyzed with a microarray for metabolic genes (“GeoChip”), producing further evidence of genomic potential for hydrocarbon degradation - genes for aerobic methane and toluene oxidation. Genes coding for anaerobic respirations, such as nitrate reduction, sulfate reduction, and metal reduction, as well as genes for carbon fixation, nitrogen fixation, and ammonium-oxidation, were also present. Our results suggest that the gabbroic layer hosts a microbial community that can degrade hydrocarbons and fix carbon and nitrogen, and has the potential to employ a diversity of non-oxygen electron acceptors. This rare glimpse of the gabbroic ecosystem provides further support for the recent finding of hydrocarbons in deep ocean gabbro from Hole 1309D. It has been hypothesized that these hydrocarbons might originate abiotically from serpentinization reactions that are occurring deep in the Earth's crust, raising the possibility that the lithic microbial community reported here might utilize carbon sources produced independently of the surface biosphere.

## Introduction

Ocean crust covers nearly 70% of the earth's surface, with an estimated volume of 10^18^ cubic meters. Microbial processes in this expansive subseafloor environment have the potential to significantly influence the biogeochemistry of the ocean and the atmosphere [1]. Recently, Delacour et al. [2] analyzed rock samples from the Atlantis Massif and reported that biomarkers were present in the gabbroic central dome (IODP Hole 1309D; this is the same drill hole we present an analysis of here) and in rocks from the Lost City Hydrothermal Field (LCHF). Delacour et al. [2] also determined that hydrocarbons were present in these basement rocks. These authors suggested that these hydrocarbons account for an important fraction of the carbon stored in the basement rocks of the Atlantis Massif.

Hydrocarbons at the Atlantis Massif are the subject of a recent report by Proskurowski et al. [3], who found that methane and other low-molecular-weight carbon compounds, which are abundant at the LCHF, appear to have formed abiotically from serpentinization reactions in olivine- and pyroxene-rich igneous rocks (peridotite). This water rock reaction evolves hydrogen [4], [5] and higher alkanes [4]. Congruent with the geochemical conditions at the LCHF Schrenk et al. [6] found a low diversity of predominantly methanogenic and/or methanotrophic Archaea in LCHF chimneys. Brazelton et al. [7] reported that LCHF carbonates and fluids are dominated by methane- and sulfur-metabolizing communities. Together, these studies suggest that LCHF carbonates host a microflora that likely utilize the rock-seawater derived electron donors/carbon sources. Thus, the precedent for hydrocarbon utilizing microbes at the Atlantis Massif has been set.

Beyond LCHF carbonates, much attention has been directed towards the basalt layer of ocean crust. Recent reports on the diversity of microbial life in marine basalts revealed that upwards of 13 or more clades of Bacteria [8]–[15] and two clades of Archaea [8], [9], [11], [12], [15] are present in this environment. Yet, little is known about the metabolic processes occurring in this environment, with only one report by Mason et al. [11] assaying for functional status of basalt microflora. Further, all but two of these studies [8], [10] were conducted on surface basalts. Thus, even in the frequently studied basalt layer little is known about subsurface endolithic microorganisms.

Our collective knowledge about endolithic microorganisms associated with igneous rocks in the marine environment stems from the aforementioned studies. To date, however, the microbiology of the intermediate layer between basalt and peridotite - the gabbro layer- has not been investigated, mostly due to the difficulty inherent in sampling the igneous portion of ocean crust, a topic that was recently reviewed by Schrenk et al. [1].

The Atlantis Massif, which is interpreted as an ocean core complex composed of deep crustal (gabbro) and upper mantle rocks (peridotite) that have been unroofed and exposed at the surface as a result of faulting [16 and references therein], [ 17], provided a rare opportunity to sample gabbros, which are generally beyond the reach of currently available drilling technologies. The goals of our study were to measure the cell density, phylogenetic diversity and metabolic diversity of endolithic microflora associated with the central dome of the Atlantis Massif. To accomplish our goals we used microscopy to determine *in situ* cell densities. Terminal restriction fragment polymorphism (T-RFLP), denaturing gradient gel electrophoresis (DGGE), cloning, and sequencing were used to assess the diversity and phylogeny of microorganisms associated with marine gabbros. Further, to provide insight into the potential metabolic diversity of the gabbroic crust microflora, we analyzed conserved regions of functional genes involved in nitrogen, carbon, sulfur, and phosphorus cycling with GeoChip, a functional gene microarray [18].

## Results

### Cell counts and contamination

Prokaryotic cell densities of interior sections of core samples from Hole 1309D over the entire 1400 m interval were below the level of detection (<10^3^ cells cm^−3^ rock), indicating that prokaryotic cell densities were extremely low in gabbroic crust. The cell density in carbonate sediment sampled in neighboring Hole U1309A during Expedition 304 (Table 1) was 1.15±0.95×10^4^ cells cm^−3^.

**Table 1:** 

Expedition	Site	Hole	Core sample	Section	Top (cm)	Botom (cm)	Depth (mbsf)	Rock type	Temperature (°C)	Alteration (%)	Bacterial 16S rDNA
304	1309	A	1(1)	2	45	58	0.45	Carbonate sediment	na	na	na
304	1309	D	na	na	na	na	∼5(2)	Water sample(3)	na	na	Y
304	1309	D	10	1	103	111	61.23	Serpentinized peridotite	14	75	Y
304	1309	D	12	2	42	50	71.69	Gabbro	14	45	Y
304	1309	D	37	2	93	101	202.83	Gabbro	17	30	Y
304	1309	D	53	1	100	111	277.4	Gabbro	21	40	Y
304	1309	D	58	1	67	73	301.07	Serpentinized peridotite	22	20	Y
304	1309	D	68	1	88	92	349.28	Gabbro	23	30	Y
304	1309	D	78	1	82	90	397.32	Gabbro	25	30	Y
305	1309	D	na	na	na	na	∼397	Water sample	na	na	Y
305	1309	D	80(4)	1	18	28	401.48	Olivine gabbro	26	10	Y
305	1309	D	82	1	27	39	410.47	Olivine-bearing Gabbro	26	10	NA
305	1309	D	90(5)	1	30	36	448.9	Olivine Gabbro	27	50	Y
305	1309	D	100	1	80	89	497.4	Olivine-rich Troctolite	29	10	N
305	1309	D	102	2	67	78	508.31	Troctolite	29	10	N
305	1309	D	122	2	76	89	604.24	Olivine-bearing Gabbro	33	30	Y
305	1309	D	133	3	122	132	658.73	Gabbro	36	30	Y
305	1309	D	142(5)	3	0	13	701.05	Gabbro	38	30	Y
305	1309	D	164	1	60	74	799.6	Gabbro	43	30	Y
305	1309	D	184	1	78	85	895.78	Olivine Gabbro	48	5	Y
305	1309	D	208	4	0	12	1004.79	Gabbro	54	1	N
305	1309	D	235	2	52	63	1131.28	Olivine-rich Troctolite	63	10	N
305	1309	D	250	1	0	11	1201.5	Olivine Gabbro	68	5	Y
305	1309	D	na	na	na	na	∼1215	Water sample	na	na	Y
305	1309	D	273	1	116	132	1313.06	Gabbro	79	20	Y
305	1309	D	290	3	136	145	1391.01	Olivine-bearing Gabbro	102	5	N

1 Carbonate sediment was collected solely for cell counts and was not examined any further.

2 Units are meters above seafloor.

3 Water samples were collected using a sterile water sampling temperature probe and served as experimental controls to determine the extent of drilling induced contamination.

4 Sample 82 was insufficient for crushing and powdering and was not analyzed further.

5 Samples assayed for functional genes by microarray.

### Microbial diversity

Low cell densities were congruent with the low species diversity in Hole 1309D samples as determined by DGGE (Expedition 304) and by T-RFLP (Expedition 305). In Expedition 304 rock samples that were collected from 0.45 to 397.32 meters below seafloor (mbsf) the same two DGGE bands were observed in all samples; therefore, microbial diversity did not change with depth, or rock type. T-RFLP analysis of Expedition 305 samples revealed that diversity did vary with depth from 401.48–1391.01 mbsf. Shannon diversity indices (H') were calculated from T-RFLP data (Methods S1) and ranged from 0 (1 peak only) to 2.1 (Figure S1). The mean Shannon value was 1.37, which represents the average value for the three different restriction enzymes used per sample (Expedition 304 samples were not considered in the diversity analysis because a different methodology was used). Diversity was correlated with rock alteration (r^2^ = 0.7), with the most altered rock (50% alteration) supporting the greatest microbial diversity (H' = 2.1) (Figure S1).

### Phylogeny

Three different phylotypes, all proteobacteria, were associated with rock samples recovered from Hole 1309D from expeditions 304 and 305. All of the rock phylotypes formed clades with uncultured and cultured microorganisms from hydrocarbon-rich environments, such as methane hydrates [19] and high temperature petroleum reservoirs [20].

One rock phylotype from expedition 304 (61.23 to 397.32 mbsf) formed a clade of closely related alphaproteobacterial sequences that included two uncharacterized isolates, the first of which was an oil-degrading microorganism isolated from seawater (GenBank acc. AM423074), the second was *Thalassospira nitroreducens*, a denitrifier isolated from seawater (GenBank acc. EF437150). Also similar to the rock phylotype was an environmental sequence from hydrothermal vent fluids [21]. Attempts to sequence a second band that was unique to the expedition 304 rock sample were unsuccessful. Five DGGE bands present in the 304 water sample, that were distinct from the bands retrieved from rock samples, were not sequenced.

**Figure 1:**
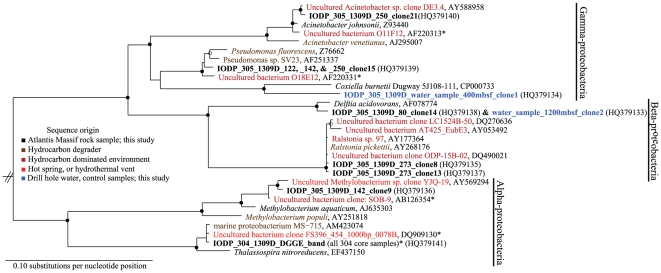
Maximum-likelihood phylogenetic tree of *proteobacterial* 16S rRNA gene sequences from Atlantis Massif samples. The environments from which microorganisms originated from are color coded (see key). Branch points supported by all phylogenetic analyses (quartet puzzling support values ≥90%) are shown by •; branch points supported by most analyses, but with less confidence (quartet puzzling support values 50–89%) are shown by ○; branch points without circles are unresolved (quartet puzzling support values <50%). Sequences <1200 nucleotides in length were inserted using the ARB parsimony insertion tool and are indicated by *. This proteobacterial ML tree was constructed with 1,000 puzzling steps. *Prochlorococcus marinus* (NC_009976) served as the outgroup (not shown).

From both rock and water samples collected during Expedition 305, a total of 480 clones were screened, 142 of which were sequenced, yielding eight unique clones (Figure 1). IODP rock clone 9 (305-1309D-142; 701.05 mbsf) was identified as an alphaproteobacterium in the *Methylobacteriaceae,* genus *Methylobacterium* (Figure 1). This clone was most similar to environmental sequences from a petroleum reservoir (GenBank acc AB126354) and from a hot spring (GenBank acc AY56924) (Figure 1). Orphan et al. [20] also reported that sequences from a high temperature petroleum reservoir branched with the *Methylobacterium*. The closest cultured organism to our clone was *Methylobacterium aquaticum* (98% similar), which was isolated from drinking water [22] (Figure 1). Although growth of *Methylobacterium aquaticum* on hydrocarbons has not been demonstrated, *Methylobacterium populi*, closely related to our clone (95% similar), was shown to grow on methane as a sole carbon and energy source [23].

IODP rock clones 8 and 13, observed in sample 305-1309D- 273 (1313.06 mbsf) were classified as *Burkholderiaceae* in the genus *Ralstonia* (Figure 1). These clones were most similar to environmental sequences from a soil column amended with phenanthrene [24], gas hydrates [19], diffuse hydrothermal fluids [25], ridge flank crustal fluids [26], and a LCHF carbonate sample [7] (Figure 1). *Ralstonia pickettii* was the most closely related cultured microorganism to rock clones (99% similar) (Figure 1). *R. pickettii* has been isolated from numerous sources such as water, soil, and activated sludge [27]. *R. pickettii* is a known hydrocarbon degrader possessing a toluene monooxygenase (Tbu), enabling it to grow on toluene, benzene, and alkylaromatics [28] (Figure 1).

IODP rock clone 21 was present in sample 305-1309D-250 (1201.50 mbsf) (Figure 1). This clone was identified as *Moraxellaceae* in the genus *Acinetobacter.* This clone was most similar to environmental sequences from deep-sea sediment (GenBank acc AY588958) and from a high-temperature petroleum reservoir [20] (Figure 1). *Acinetobacter johnsonii* was the most closely related cultured microorganism (99% similar) (Figure 1). *A. johnsonii*, which is widely distributed in the environment [29], has not been shown to grow on hydrocarbons; however, several *Acinetobacter* are known hydrocarbon-degraders, for example, *Acinetobacter venetianus* (96% similar), utilizes C_10_ to C_40_ alkanes for growth [30].

IODP rock clone 15 was classified as *Pseudomonadaceae* in the genus *Pseudomonas* (Figure 1). This phylotype was present in samples 305-1309D-122, -142, and -250 (604.24, 701.05, and 1201.50 mbsf). It was most closely related to an uncharacterized *Pseudomonas* isolated from oil-contaminated soil, that possesses plasmid-bearing genes that code for toluene (and xylene) metabolism [31]. Rock clone 15 was also similar to an environmental clone from a high-temperature petroleum reservoir [20] (Figure 1). The most closely related characterized organism was *Pseudomonas fluorescens* (98% similarity; Figure 1), which is both abundant and widely distributed in the environment [19]. AlkB genes (which oxidizes alkanes C_5_–C_12_) [32] were amplified from *Pseudomonas fluorescens* when grown on hydrocarbons as substrate [33] (Figure 1).

IODP rock clone 14 was most similar to a clone from the 1200 mbsf water sample (water sample-1200 mbsf-clone2) (Figure 1). This rock clone was closely related to *Delftia acidovorans*. Clones from rock samples that were most similar to water sample microflora were defined as contaminants. IODP clone 14 originated from sample 305-1309D-80, which was the first core collected during 305 (water was pumped into Hole 1309D at the end of Expedition 304. Drilling recommenced ∼2 weeks later during Expedition 305). Given the presence of a water sample microorganism in a rock sample (80), it is highly likely that contamination occurred during the intervening period between IODP expeditions 304 and 305, or at the onset of drilling during 305. Also identified in a water sample was a clone (water sample-400 mbsf-clone 1) most closely related to *Coxiella burnetii* (GenBank acc CP000733).

### Functional genes

Of the 24,243 probes present on the GeoChip microarray [18], 103 were positive in rock sample 90 (448.90 mbsf) and 165 were positive in rock sample 142 (701.05 mbsf) (Table S1). Results of the microarray analysis revealed that genes coding for hydrocarbon-degradation were present (Table 2; Table S1), which provides support for the metabolic functions inferred from phylogenies (Figure 1). Beyond hydrocarbon degradation, numerous other functional genes coding for processes such as carbon fixation, nitrogen fixation, ammonium-oxidation, nitrate reduction, organic contaminant degradation, and metal reduction/resistance were observed (Table 2; Table S1).

**Table 2:** Relative abundance of functional genes from IODP Expedition 305 rock samples 90 and 142.

Gene Category	Rock sample 90 (% relative abundance)	Rock sample 142 (% relative abundance)
Organic contaminant degradation	34.19	44.62
Metal toxicity	16.13	18.73
Carbon degradation^(*)^	14.19	7.17
Denitrification^(*)^	10.97	6.37
Nitrogen mineralization^(*)^	3.23	2.39
Sulfate reduction^(*)^	4.52	4.38
Nitrogen fixation^(*)^	4.52	1.99
Carbon fixation^(*)^	3.23	2.39
Methane oxidation^(*)^	3.23	1.99
Cytochrome	1.94	2.79
Methane generation	0.65	0.80
Nitrification^(*)^	0.65	0.00

Gene categories that have a higher relative abundance in rock sample 90 compared to rock sample 142 are denoted by*.

The predominant genes in both rock samples coded for organic contaminant degradation, carbon cycling, nitrogen cycling, and metal toxicity (Table 2). Of the genes involved in carbon cycling the majority coded for carbon degradation, followed by carbon fixation, methane-oxidation, and methane generation (Table 2). Genes coding for denitrification accounted for the majority of genes involved in nitrogen cycling with fewer genes observed that code for nitrification, nitrogen fixation, and nitrogen mineralization (Table 2). The remaining genes coded for sulfite reduction, and iron-reduction (cytochrome) (Table 2).

The proportions of genes detected in the functional gene categories differed between sample 90 and 142 (Table 2), which was collected ∼252 meters deeper into the central dome. In the deeper sample the functional gene categories organic contaminant degradation, metal toxicity, metal reduction, and methane generation had higher relative abundances (Table 2).

### Isotopes

**Figure 2:**
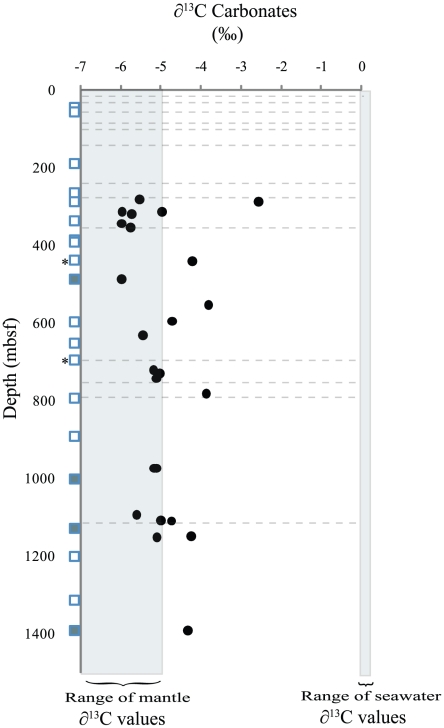
Carbon isotopes from Atlantis Massif samples. Carbonate ∂13C isotopic values were measured at various depths over the 1400 mbsf interval. Faults are indicated by dashed lines. Samples collected for microbiological analyses are indicated by squares to the left of the graph of carbonate ∂13C isotopic values; unfilled squares indicate that ribosomal DNA was successfully amplified from a sample; solid squares indicate that no ribosomal DNA was amplified. Samples that were analyzed by microarray to assay for functional genes are denoted by *.

δ^13^C values were determined in rocks over the entire 1400 mbsf interval (Figure 2). Our analysis of the isotopic signature recorded in carbonate in 1309D rocks revealed δ^13^C values ranging from −2.9 to −6.0 ‰ (Figure 2), with an average of −5.0‰.

## Discussion

### Cell density

Cells counts from the rock samples recovered by drilling were below the limit of detection (<10^3^ cells cm^−3^ rock). Cell densities much lower than those reported for basalts and LCHF carbonates and crustal fluids were detected in sediment sampled next to our sample site, providing support for our fining that cell densities within our subsurface rock samples were extremely low (below the level of detection). For example, the cell density in carbonate sediment sampled in neighboring Hole U1309A collected during Expedition 304 (Table 1) was 1.15±0.95×10^4^ cells cm^−3^, far below values reported for basalts - up to 10^6^ [34], and 10^9^ cells per g rock [14]. Carbonates from LCHF host up to 10^8^ cells per g dry weight [6] and 10^9^ cells per g rock [7].

### Microbial diversity

Recently, Orcutt et al. [35] used T-RFLP to evaluate two basalts that were identified by Santelli et al. [14] as low and high diversity samples from Loihi Seamount. Orcutt et al. [35] reported Shannon values (H') of 1.81 (low diversity sample) and 2.55 (high diversity sample). In comparison to these basalt samples Expedition 305 rocks appear to harbor a lower diversity of microorganisms, with an average Shannon value of 1.37, than the least diverse basalt sample. Interestingly, the depth variance in microbial diversity in Expedition 305 rock samples was related to percent rock alteration (Figure S1). Samples with higher percent alteration had a greater diversity of microorganisms (Figure S1). Alteration could result in, for example, changing rock permeability and oxidation state of the rocks, which may affect the microbial communities, by providing additional niches in gabbroic rocks. This potential niche expansion may be reflected by higher microbial diversity in the more altered rocks.

### Phylogeny

Perhaps surprisingly, the gabbro microbial communities we observed do not appear to be endemic to ocean crust. Marine basalts and gabbros are nearly identical in chemical composition, thus we had hypothesized that similar communities of endolithic microorganisms specialized for growth in subsurface igneous rocks would be recovered from gabbros. Our analysis of gabbroic rocks revealed that there was no overlap in the microbial communities between these two rock types - none of the ocean crust clades that appear to be endemic to basalts that were delineated by Mason et al. [12] were found in gabbros. The absence of clades determined to be endemic to ocean crust within marine gabbros in conjunction with the widespread distribution in the environment (e.g. water, soil, and activated sludge) of cultured representatives that are closely related to our clones suggests that gabbroic microflora are not specifically adapted to life in ocean crust.

Second, one would expect that microorganisms endemic to ocean crust (e.g. gabbro) would have 16S rDNA sequences that have diverged from related microbes that are not endemic to this habitat, inclusive of cultured and uncultured representatives from non-crustal environments. This was observed in Mason et al. [12], in which several clades that appear to be endemic to basalt were not closely related to any cultured microorganisms, or to microbes found in seawater, or other non-crustal environments. The close relationship of gabbroic microorganisms to cultured hydrocarbon degrading Bacteria that are widely distributed in the environment suggests that gabbroic microflora are transient microorganisms that are not endemic to ocean crust. The high similarity of gabbroic microflora to both cultured and uncultured representatives previously reported in hydrocarbon dominated environments suggests instead that gabbroic microflora are not ocean crust specialists, such as those observed in basalts, but rather predominate in environments where hydrocarbons are present.

The high similarity of 16S rRNA gene sequences of gabbroic endoliths to cultured representatives was surprising, yet not unprecedented in other hydrocarbon dominated environments, such as gas hydrates [19] and petroleum reservoirs [20] (sequences from Expedition 305 rocks were highly similar to clones from both of these studies). In fact, Lanoil et al. [19] noted the high similarity of the Bacteria (ca. 72%) in a gas hydrate sample that were related at the species level to cultured microorganisms as an unusual feature of the bacterial diversity in this hydrocarbon dominated environment. Similar to the results presented by Lanoil et al. [19] the Bacteria observed in high temperature petroleum reservoirs were 97–100% similar to previously cultured representatives [see Table 2 in 20]. More recently clones from crustal fluids [26] and from LCHF (∼5 km from our study site) carbonate chimneys and from vent fluids [7] were 100% similar to *Ralstonia pickettii* and to clones from rock sample 273 (1313.06 mbsf). Although the high similarity of microorganisms to previously cultured hydrocarbon degrading bacteria does not necessarily mean that the *in situ* gabbroic community shares the same genetic potential as their close relatives, the data does suggest that within disparate hydrocarbon dominated environments certain bacterial taxa are generalists, able to survive and to potentially degrade hydrocarbons in a myriad of environments, including deep subsurface igneous rocks, such as those analyzed in this study.

### Biogeochemical cycling

In congruence with the close phylogenetic relationships of rock associated microorganisms to those from hydrocarbon-dominated environments and to known hydrocarbon degrading Bacteria, genes coding for hydrocarbon degradation were observed in both rock samples. For example, genes coding for aerobic methane-oxidation (*pmo* and *mmo*) were observed in rock samples 90 and 142 (Table S1). *Methylobacterium populi*, closely related to clones in rock sample 142 (95% similar), was shown to grow on methane as a sole carbon and energy source [23]. Additionally, genes coding for aerobic toluene oxidation were present in rock sample 142 (Table S1). Specifically, genes coding for toluene oxidation from *Pseudomonas mendicina* were present (Table S1). Clones in rock sample 142 that were most closely related to *Pseudomonas fluorescens*, which is able to grow anaerobically on toluene [36], were also highly similar to *P. mendicina* (96% similar). Taken together phylogenetic and functional gene analyses converge to suggest that hydrocarbon oxidation may be occurring in deep subsurface ocean crust.

Beyond hydrocarbon oxidation, many other functional genes involved in carbon cycling were present (Table S1). For example, genes coding for carbon fixation (*acsA*, FTHFS, rbcL, rbcS) were present in both rock samples. Delacour et al. [2] reported that total organic carbon (TOC) ranged from 53–1015 ppm. TOC concentrations in rocks above and below sample 90 (448.90 mbsf) are lower than the TOC concentrations in rocks near sample 142 (701.05 mbsf) [see Table 1 in 2]. Interestingly, the relative abundance of genes coding for carbon fixation were slightly lower in rock sample 142 (2.4%) than in sample 90 (3.2%). This suggests that in local environments within ocean crust where organic carbon is low relative to other sections of the crust there is a genetic potential, in the form of genes coding for carbon fixation, to offset lower TOC concentrations by an increase in carbon fixation.

*Mcr* genes coding for methane production were identified in both rock samples (Table S1). Interestingly, the alpha subunit (*mcrA*) of the methyl conenzyme M reductase (MCR) from an anaerobic methane oxidizing archaea (ANME) was present in sample 142 [37] (Table S1). Although *mcr* genes were present in our rock samples, no Archaea were observed in either rock or seawater samples despite numerous attempts to amplify archaeal 16S rRNA genes (see Methods); therefore, it is unlikely that Archaea play a significant role in biogeochemical cycling in the marine gabbros analyzed here.

Genes coding for denitrifying processes (e.g. *nar*G, *nir*K, *nor*B) were detected in both rock samples (Table S1). Although the majority of characterized hydrocarbon-degrading microorganisms previously discussed are aerobic, both *R. picketti* [38] and *P. fluorscens* [36] have been shown to oxidize hydrocarbons by denitrification. This suggests that hydrocarbons be may oxidized anaerobically in the central dome of the Atlantis Massif.

Analysis of metagabbros revealed that nitrogen concentrations are low (4.0 to 4.5 ppm) [39]. Thus nitrogen fixation in this environment would be paramount. Nitrogen-fixation in the marine subsurface was recently reported in hydrocarbon dominated seep sediments [40]. Further Mason et al. [11] reported that genes coding for nitrogen-fixation were present in a Juan de Fuca basalt. The presence of genes coding for nitrogen fixation in our gabbroic samples (Table S1), in conjunction with the findings of Mason et al. [11] and Dekas et al. [40], suggests that this process may be widespread in the marine subsurface. Previously unrecognized sites for nitrogen-fixation in the marine environment, such as subsurface rocks, may provide insight into the missing nitrogen sources in the ocean as presented by Deutsch et al. [41].

Other genes that were observed code for dissimilatory sulfate reduction (*dsrA* and *dsrB*) (Table S1), largely from uncultured sulfate-reducing bacteria, which may suggest that hydrocarbons are degraded anaerobically by sulfate reducers [42]. Further, genes coding for cytochromes from, for example, *Geobacter sulfurreducens,* a metal- and sulfur-reducing Bacteria isolated from a hydrocarbon contaminated environment [43] were detected in both rock samples (Table S1).

The presence of genes coding for both aerobic and anaerobic respiration in the upper 700 meters of Hole 1309D are consistent with the redox conditions suggested by Delacour et al. [44] who reported that strontium and sulfur isotopes are elevated towards seawater values in the upper 800 m in 1309D. These isotopic values indicate that seawater has circulated in the upper portion of the central dome [45] and are correlated with a greater degree of serpentinization [2]. Seawater circulating within the top 800 m would provide a limited amount of oxygen that is required for aerobic processes, with a transition to anaerobic processes following oxygen depletion. Below 800 m these authors suggested that reducing conditions prevail and that seawater circulation is constrained to faults within the central dome.

### *In situ* hydrocarbons

Our analysis of the isotopic signature recorded in carbonate in Hole 1309D rocks revealed δ^13^C_carbonates_ averaged −5.0‰. Mantle carbon δ^13^C ranges from approximately −5.0 to −7.0‰ [2], [46], with the majority of our samples falling in this range (Figure 2). Delacour et al. [2] reported that *n*-alkanes ranging from C_15_ to C_40_ (volatiles could not be measured) were present in rocks from the central dome. These alkanes were unbranched, with no carbon number predominance, and showed a decrease in abundance with increasing carbon number [2]. This profile is similar to carbon compounds synthesized abiotically by Fischer-Tropsch type (FTT) reactions [47], such as at those at the LCHF [3]. Abiotic production of the unbranched alkanes (which would include methane) in Atlantis Massif samples is suggested. These hydrocarbons could provide carbon and energy to extant microbes in the interior of the Atlantis Massif.

In 1309D rocks Delacour et al. [2] also identified the biomarkers squalane, hopane, sterane, pristane, and phytane. These alkanes were attributed to DOC input from seawater circulating throughout the Atlantis Massif [2]. The source of these alkanes may reflect input of marine DOC as these authors suggest, alternatively squalene (diagenetically transformed to squalane [2]) has been identified in methanotrophs [48] and hopanes are found in a variety of prokaryotes [49], including methanotrophs [50]. Steranes are ubiquitous in eukaryotes [51], and although rare in prokaryotes, have been reported in a few microorganisms such as methanotrophs in the *Methylococcales* [48], [52]. Pristane and phytane could originate from methanogenic Archaea [53]. The δ^13^C values of individual biomarkers were not determined in Atlantis Massif samples; therefore, their exact origin is not known but the results of our molecular analyses indicates that *in situ* microorganisms, and in particular methanotrophs, are the sources of biomarkers in 1309D.

### Conclusion

Our results raise the intriguing possibility that hydrocarbons in very deep ocean rocks support microbial communities. Additionally, we show that the genetic potential for novel metabolic processes, such as carbon and nitrogen fixation, is present within an unexplored layer of ocean crust. Our findings, particularly regarding the presence of genes coding for methane cycling, have implications not only for Earth's subsurface, but also for other planets such as Mars. Methane on Mars is concentrated in some equatorial regions of the atmosphere, which suggests that it is derived from localized geological sources [54]. Although the exact mechanism by which methane forms on Mars is not known, serpentinization reactions in the Martian subsurface have recently been proposed [55]. Therefore, similar to the Atlantis Massif, the Martian subsurface may harbor methane-consuming prokaryotes. Future efforts should be directed towards quantifying the role endolithic prokaryotes play in methane cycling and in determining the sources of methane, and other hydrocarbons in marine crust. These findings will undoubtedly focus attention on obtaining more information on the geochemistry of formation fluids from deep ocean rocks, which are technically challenging to acquire requiring different sampling technologies than those used in the design of this exploratory study.

## Methods

### Sampling

**Figure 3:**
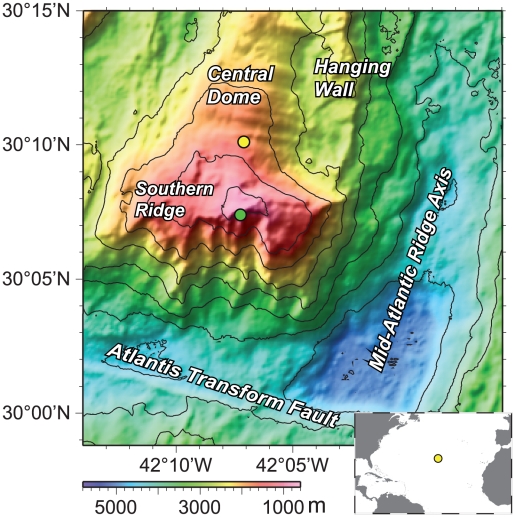
Map of the Altantis Massif showing the locations of the Integrated Ocean Drilling Program Expeditions 304 and 305, Hole 1309D (yellow circle) and the Lost City Hydrothermal Field (green circle). Inset figure shows the location of the Atlantis Massif (yellow circle), where Hole 1309D and the Lost City Hydrothermal Field are located. Figure adapted from Blackman, D. K., J. A. Karson, D. S. Kelley, J. R. Cann, G. L. Früh-Green, J. S. Gee, S. D. Hurst, B. E. John, J. Morgan, S. L. Nooner, D. K. Ross, T. J. Schroeder and E. A. Williams (2002). “Geology of the Atlantis Massif (Mid-Atlantic Ridge, 30°N): Implications for the evolution of an ultramafic oceanic core complex.” Marine Geophysical Researches 23(5): 443–469.

For this study, the gabbroic central dome of the Atlantis Massif (30°10.120′N, 42°7.113′W, ∼15 km from the Mid-Atlantic Ridge) was sampled at Hole 1309D by drilling during the Integrated Ocean Drilling Program (IODP) Expedition 304 (0–400 mbsf) and Expedition 305 (400–1400 mbsf) (Figure 3, Table 1). Twenty-two mainly gabbroic rock samples were collected specifically for microbiological analyses (Table 1). The temperature range from which samples were collected was 14 to 102°C(Table 1). Further, seawater and borehole water, which served as experimental controls, were collected with a sterile water sampling temperature probe at five meters above the seafloor, at 397 mbsf, and at 1215 mbsf (Table 1). Hole 1309D temperature and percent alteration values were obtained as described by Blackman et al. [56]. Rock samples intended for molecular analyses were maintained at −80°C until the time of analysis.

### Cell counts

To obtain cell counts for Expedition 304 samples the interior portions of rock samples was added to filter-sterilized phosphate-buffered saline solution (130 mM NaCl, 10 mM NaPO4, pH 7.2) with 10 µg/ml 4′,6-diamidino-2-phenylindole dihydrochloride (DAPI). Samples were mixed vigorously with a vortexer for 30 min. Supernatant was filtered with a 0.2 µm filter.

For Expedition 305 samples crushed, interior portions of rock samples were fixed with 6 ml paraformaldhyde (4%w/v) to which 600 µl sodium pyrophosphate (0.1 M) was added. Samples were then agitated for three hours on a vortexer to detach cells from rock material. After vortexing, samples were stored for 12 hours at 4°C and subsequently stained with a 0.2 µm filter sterilized acridine orange (AO) (0.01%w/v) solution and filtered onto a 0.2 µm filter. Both 304 and 305 rock samples were analyzed using a Zeiss Axiophot microscope.

### Nucleic acid extraction and whole genome amplification

For shorebased molecular analyses 304 and 305 core samples were processed as previously described Fisk et al. [8]. Up to four grams of interior, powdered, rock sample was extracted using a Mo-Bio Soil DNA extraction kit (Mo Bio Laboratories, Carlsbad, CA, USA) according to the manufacture's protocol. Negative DNA extractions were processed in parallel to rock and water sample extractions. Genomic DNA from rocks, negative DNA extractions, and water samples from 305 were amplified using a Qiagen REPLI-g® Midi Kit (Germantown, MD, USA), following the manufacturer's protocol.

An additional attempt was made to elicit Archaea from Expedition 305 rock samples 90 and 142 (these samples were analyzed using the GeoChip microarray) by 1) increasing the volume of extracted rock material to 10 g using a Mo Bio UltraClean Mega Soil DNA Isolation Kit and by 2) modifying the manufacturer's protocol. Specifically, following addition of the first solution, samples were incubated at 65°C for 5 min, vortexed briefly, and placed at 65°C for 5 min for a second time. After adding the Mo Bio IRS solution samples were placed at 4°C for 5 min. Genomic DNA from these samples were amplified using the Qiagen REPLI-g® Midi kit.

### Drilling related contamination

To determine the extent of contamination from drilling fluid, 0.5 µm fluorescent microspheres were deployed in a plastic bag wedged into the core catcher that ruptured as the first cored material entered the core barrel. Each core collected was rinsed and assessed by microscopy (Zeiss Axiophot, Thornwood, NY, USA) to ensure that microspheres were dispersed. Contamination of the interior of the core was determined by the presence or absence of microspheres. In addition, water samples were obtained using a sterile water sampling temperature probe (WSTP). These samples served as molecular controls to better constrain the degree of contamination. The WSTP water collection device was initially flushed with distilled water and then sterilized with a (10%v/v) bleach solution, which remained in the tool for 0.5 h. The tool was then rinsed with nanopure water. Approximately 5 ml of seawater was collected and immediately frozen at −80°C.

Microspheres dispersed during drilling operations to constrain drilling induced contamination were observed on the exterior, but not in the interior of all core samples. This suggests that drilling fluids did not contaminate samples. Although microspheres were not observed in the interior of any rock samples, our molecular analysis revealed that rock sample 80, the first core collected during Expedition 305, contained microorganisms closely related to *Delftia acidovorans*. *D. acidovorans* was also found in the water sample from 1200 mbsf; therefore, we suspect that the drilling process may have contaminated this core sample and it was not further analyzed.

### PCR, clone library construction, DGGE, T-RFLP, RFLP, and sequencing

For DGGE analysis of 304 core samples DNA fragments encoding bacterial 16S rRNA genes were amplified in a TaKaRa Ex Taq (Takara Bio, Otsu, Japan) PCR cocktail (final concentration 1X) with 0.25 µM (final concentration) of the primers Eub341F with a GC-clamp (5′-CGC CCG CCG CGC CCC GCG CCC GTC CCG CCG CCC CCG CCC GCC TAC GGG AGG CAG CAG-3′), and Univ907R (5′-CCG TCA ATT CMT TTR AGT TT-3′) [57]. Amplifications were carried out in a iCycler thermal cycler (Bio-Rad Laboratories, Hercules, CA, USA) with the following conditions: an initial denaturation step of 94°C for 5 min and then 30 cycles of 94°C for 20 s, 54°C for 20 s, and 72°C for 2 min. DGGE was performed with D-code systems (Bio-Rad Laboratories) with 6% (wt/v) polyacrylamide gel with denaturing gradients from 20 to 60% (100% denaturant: 7 M urea and 40% v/v deionized formamide) at 200 volts at 60°C for 4 h. DGGE of reamplified PCR products was used to check band purity. PCR amplifications were purified with QIAquick PCR purification kit (QIAGEN Valencia, CA, USA) and used as the DNA template for sequencing. With the same conditions used for bacterial 16S rDNA amplifications the primers Arch344F with a GC-clamp (5′-CGC CCG CCG CGC CCC GCG CCC GTC CCG CCG CCC CCG CCC GAC GGG GYG CAG CAG GCG CGA-3′) and Arch915R (5′-GTG CTC CCC CGC CAA TTC CT-3′) [58] were used to assay for Archaea.

For 305 core samples bacterial 16S rDNA were amplified from rock samples and water samples in 2X PCR Master Mix (Fermentas, Glen Burnie, MD, USA) (final concentration 1X) with 0.5 µM final concentration of 27F-B (5′-AGRGTTYGATYMTGGCTCAG-3′) and 1492R (Lane, 1991). Amplifications were carried out in a PTC-200 Thermal Cycler (MJ Research, Watertown, MA, USA) with the following conditions: 35 cycles of 94°C for 15 s, 55°C for 1 min, and 72°C for 2 min, with a final extension of 72°C for 5 min. T-RFLP reactions were carried out with the same conditions as above except the 27F-B was 5′ end labeled with the phosphoramidite fluorochrome 5-carboxy-fluorescein (6-FAM). T-RFLP products were digested in three separate aliquots with *Bsu*I, *Alu*I, and *Hin*6I (Fermentas) overnight at 37°C.

16S rRNA genes amplified from genomic DNA from Expedition 305 rock samples 80, 90, 122, 142, 250, and 273 were pooled and cloned. To identify the sample of origin for rock clones, cloned inserts were amplified with a fluorescently labeled forward primer. PCR products were digested with three different restriction enzymes and compared to the sample T-RFLP profiles.

Clone library construction, screening and processing were carried out as previously described [12]. Briefly, bacterial amplification products were cloned into pGEM-T Easy Vector (Promega, Madison, WI, USA) and 16S rDNA inserts were amplified with M13 primers. Full-length inserts were characterized by restriction fragment length polymorphism (RFLP) analysis. The inserts of the first 96 clones were digested with the restriction enzymes *Bsu*R1 (*Hae*III) and *Alu*I (Fermentas) overnight at 37°C with the appropriate buffer and 10 units of enzyme. Few additional clones were discerned with *Bsu*R1 (*Hae*III); therefore the remaining 384 clones were digested with *Alu*I only. Digested PCR products were resolved on a 3% agarose gel. One clone from each unique RFLP pattern was sequenced with M13F on an ABI 3730 capillary sequencer. M13R was used to generate near full-length sequences for several clones representing each phylotype. Chimeric sequences were identified with Pintail [59] and Mallard [60]. Archaea were assayed using the primer pairs 20F [61] and 1492R, 20F and 1406R [62], 20F and 519R [63] with the same PCR conditions used to amplify bacterial 16S rDNA, except 40 cycles were used. Additionally, semi-nested PCR reactions using 20F/1492R and 20F/1406R amplifications as template were carried out similar to bacterial 16S rDNA reactions, with 30 cycles instead of 35. Finally, the archaeal primers 8F and 958R [64] were used to amplify archaeal 16S rRNA genes from genomic DNA from rock samples 90 and 142 that was extracted using a Mo Bio UltraClean Mega Soil DNA Isolation Kit using a modified protocol and amplified with Qiagen REPLI-g® Midi kit.

### Nucleotide sequence accession numbers

Bacterial 16S rDNA sequences generated for this study were submitted to the GenBank database under the accession numbers HQ379133- HQ379141.

### Phylogenetic analysis

Phylogenetic analysis was carried out as described by Mason et al. [12]. Briefly, Ribosomal RNA gene sequences from rock and water samples were searched against GenBank [65] and similar sequences were imported and aligned in ARB [66]. Near full-length sequences, consisting of at least 1200 nucleotides, were used to construct neighbor-joining, parsimony, and maximum-likelihood phylogenetic trees. Maximum-likelihood trees of near full-length sequences were generated in ARB [66] using Tree-Puzzle [67] with the Hasegawa-Kishino-Yano model [68]. Shorter sequences were added to maximum-likelihood trees using the ARB parsimony insertion tool [69].

### Functional gene analysis

Functional genes were assayed for using the GeoChip 2.0 [18] microarray following previously described methods [70]. Briefly, amplified DNA from two core samples, 305_1309D_90 and _142 was amplified in triplicate using a Templiphi 500 amplification kit (Amersham Biosciences, Piscataway, NJ, USA) with modifications as previously described [70]. Amplified DNA was fluorescently labeled with Cy5. Hybridizations were performed using an HS4800Pro Hybridization Station (TECAN, US, Durham, NC, USA) overnight at 42°C. Microarrays were scanned using a ProScanArray (PerkinElmer, Waltham, MA, USA). Images were then analyzed using ImaGene 6.0 (BioDiscovery, El Segundo, CA, USA) to designate the identity of each spot and to determine spot quality. Data was processed as described by Wu et al. [70]. Protein functions were determined by searching the Universal Protein Resource (UniProt) database [71].

### Isotopes

Clean, handpicked calcite splits were used for isotopic analysis of carbonates. The carbon isotope composition of calcites was analyzed at GeoForschungsZentrum Potsdam in continuous flow mode with a Finnigan GasBench II (Thermo Fisher Scientific, Inc., Waltham, MA, USA) connected with a DELTAplusXL (Thermo Finnigan, Bremen, Germany) mass spectrometer. From each sample, ca 0.25 mg was loaded into 10 ml Labco Exetainer® vials. After automatically flushing with helium, the samples were reacted in phosphoric acid (100%, density 1.93) at 75°C for 60 min in a Finnigan GasBench preparation system, as previously described [72]. Carbon isotope compositions were given relative to the VPDB standard (Pee Dee Belemnite marine carbonate standard) in the conventional d^13^C-notation, and were calibrated against three international reference standards (NBS 19, CO1, CO8). The standard deviation (1 sigma) for both standard and duplicate analyses was 0.06‰.

## Supporting Information

Figure S1 Shannon diversity indices of T-RFLP data from Integrated Ocean Drilling Program Expedition 305 rock samples and percent rock alteration.

Table S1 List of functional genes from Expedition 305 rock samples 90 and 142.

Methods S1
