# Supplementary material for: First Investigation of the Microbiology of the Deepest Layer of Ocean Crust
Source: PLoS One. 2010 Nov 5;5(11):e15399. doi: 10.1371/journal.pone.0015399 (PMC2974637; doi:10.1371/journal.pone.0015399)
Supplement: Table S1 — List of functional genes from Expedition 305 rock samples 90 and 142. [file pone.0015399.s002.pdf]

Table S1. List of functional genes from Expedition 305 rock samples 90 and 142.

Rock sample 90

Genbank ID	Gene short description	Gene category	Organism
662361	cellulase	CDEG	Pectobacterium carotovorum
38636430	cellulase	CDEG	Neurospora crassa
15839440	cellulase	CDEG	Mycobacterium tuberculosis CDC1551
20806951	cellulase	CDEG	Thermoanaerobacter tengcongensis
8489861	cellulase	CDEG	Thermobifida fusca
22985743	chitinase	CDEG	Burkholderia fungorum
29655324	chitinase	CDEG	Coxiella burnetii RSA 493
27348228	chitinase	CDEG	Nocardiopsis prasina
2429326	chitinase	CDEG	Stenotrophomonas maltophilia
11995035	chitinase	CDEG	Burkholderia cepacia
30793461	chitinase	CDEG	Paenibacillus ehimensis
23472783	chitinase	CDEG	Pseudomonas syringae pv. syringae B728a
2952289	chitinase	CDEG	Stenotrophomonas maltophilia
26249933	chitinase	CDEG	Escherichia coli CFT073
23305801	laccase	CDEG	Phaeosphaeria spartinicola
1100246	laccase	CDEG	Trametes villosa
10441362	laccase	CDEG	Ganoderma lucidum
2832600	mannanase	CDEG	Rhodothermus marinus
28200469	mannanase	CDEG	Cellvibrio japonicus
15827228	pgl	CDEG	Mycobacterium leprae TN
34366094	polygalacturonase	CDEG	Aspergillus aculeatus
17549101	polygalacturonase	CDEG	Ralstonia solanacearum GMI1000
22987745	0	CFIX	Rhodobacter sphaeroides
25900636	FTHFS	CFIX	Desulfomicrobium baculatum
25293736	FTHFS	CFIX	Streptococcus pneumoniae
25900626	FTHFS	CFIX	Desulfovibrio piger
567190	rbcS	CFIX	manganese-oxidizing bacterium SI85-9A1
12667574	dsrA	DSR	uncultured sulfate-reducing bacterium UMTRAdsr828-17
7262420	dsrA	DSR	uncultured sulfate-reducer HMS-54
7262428	dsrA	DSR	uncultured sulfate-reducer HMS-24
34604741	dsrA	DSR	uncultured sulfate-reducing bacterium
FW015015B	dsrB	DSR	lab clone
TPB16070B	dsrB	DSR	lab clone

15077478	dsrB	DSR	Desulfofaba gelida
3095051	arsenic	MET	Pseudomonas aeruginosa
23102528	arsenic	MET	Azotobacter vinelandii
17937251	arsenic	MET	Agrobacterium tumefaciens str. C58
25027431	arsenic	MET	Corynebacterium efficiens YS-314
21233460	cadmium	MET	Xanthomonas campestris pv. campestris str. ATCC 33913
27468433	cadmium	MET	Staphylococcus epidermidis ATCC 12228
6466219	cadmium,zinc,cobalt	MET	Zymomonas mobilis
23055124	chromium	MET	Leuconostoc mesenteroides subsp. mesenteroides ATCC 8293
21230722	cobalt,zinc,cadmium	MET	Xanthomonas campestris pv. campestris str. ATCC 33913
14331038	copper	MET	Pseudomonas fluorescens
37222119	copper	MET	uncultured bacterium
17937687	copper	MET	Agrobacterium tumefaciens str. C58
728691	copper	MET	Saccharomyces cerevisiae (baker's yeast)
23475584	cytochrome	MET	Desulfovibrio desulfuricans G20
24372200	cytochrome	MET	Shewanella oneidensis MR-1
2865531	cytochrome	MET	Shewanella putrefaciens
32477238	lead	MET	Pirellula sp. 1
38016798	lead	MET	Klebsiella pneumoniae
24372984	mercury	MET	Shewanella oneidensis MR-1
13472464	mercury	MET	Mesorhizobium loti MAFF303099
6689526	mercury	MET	Xanthomonas campestris
20095139	mercury	MET	Providencia rettgeri
8575576	nickel	MET	Wautersia eutropha
22988087	tellurium	MET	Rhodobacter sphaeroides
16120989	tellurium	MET	Yersinia pestis CO92
16761960	tellurium	MET	Salmonella enterica subsp. enterica serovar Typhi str. CT18
38016657	tellurium	MET	Klebsiella pneumoniae
1749680	zinc,cadmium	MET	Schizosaccharomyces pombe (fission yeast)
13259303	mcrA	methane_gen	uncultured methanogen RS-ME32
28070885	mmo	methane_ox	Methylosinus trichosporium
2098698	mmoA	methane_ox	Methylocystis sp. M
22324411	pmoA	methane_ox	uncultured bacterium
37496853	pmoA	methane_ox	uncultured bacterium
34017074	pmoA	methane_ox	uncultured bacterium
29293188	nifH	NFIX	uncultured bacterium

1255456	nifH	NFIX	unidentified bacterium
19070843	nifH	NFIX	unidentified nitrogen-fixing bacteria
3157506	nifH	NFIX	unidentified nitrogen-fixing bacteria
1255484	nifH	NFIX	unidentified bacterium
3157528	nifH	NFIX	unidentified nitrogen-fixing bacteria
23867892	nifH	NFIX	unidentified nitrogen-fixing bacteria
14009569	amoA	NIT	uncultured bacterium gp22
26665349	hao	NIT	Nitrosospira multiformis
13474124	urease	NIT	Mesorhizobium loti MAFF303099
15807979	urease	NIT	Deinococcus radiodurans R1
23011399	urease	NIT	Magnetospirillum magnetotacticum
33864494	urease	NIT	Prochlorococcus marinus str. MIT 9313
1174887	urease	NIT	Staphylococcus xylosus
12313640	urease	NIT	Rhodobacter capsulatus
37731823	urease	NIT	Nitrosomonas cryotolerans
2440024	urease	NIT	Wautersia eutropha
26278794	narG	NRED	uncultured bacterium
32307919	narG	NRED	uncultured bacterium
24983618	nasA	NRED	Pseudomonas putida KT2440
34103538	nasA	NRED	Chromobacterium violaceum ATCC 12472
30269589	nasA	NRED	uncultured bacterium
2982930	nasA	NRED	Aquifex aeolicus VF5
19072877	nirK	NRED	Pseudomonas mendocina
22252874	nirS	NRED	uncultured bacterium
28542639	nirS	NRED	uncultured bacterium
37999158	nirS	NRED	uncultured bacterium
11344610	nirS	NRED	Pseudomonas fluorescens
29466004	norB	NRED	uncultured bacterium
29465998	norB	NRED	uncultured bacterium
38373187	nosZ	NRED	uncultured bacterium
3057083	nosZ	NRED	Paracoccus pantotrophus
18643736	nosZ	NRED	Azospirillum sp. A1-3
4633572	nosZ	NRED	uncultured bacterium ProR
17298107	2,4-D	ORG	Bradyrhizobium sp. HW13
22002454	2,4-D	ORG	uncultured bacterium
40890245	acrylonitrile	ORG	uncultured organism

27657371	acrylonitrile	ORG	uncultured bacterium Dr1
18976382	aminocyclopropane	ORG	Pyrococcus furiosus DSM 3638
22126223	atrazine	ORG	Yersinia pestis KIM
23450983	benzoate-anaerobic	ORG	Thauera aromatica
23119300	benzoate-anaerobic	ORG	Desulfitobacterium hafniense
3925514	benzoate-anaerobic	ORG	Pseudomonas aeruginosa
2190582	benzoate-anaerobic	ORG	Rhodopseudomonas palustris CGA009
23114437	benzoate-anaerobic	ORG	Desulfitobacterium hafniense
30022110	benzoate-anaerobic	ORG	Bacillus cereus ATCC 14579
33595881	benzoate-anaerobic	ORG	Bordetella parapertussis 12822
218366	benzoate-anaerobic	ORG	Candida tropicalis
4126496	benzonitrile	ORG	Rhodococcus sp. N-771
3172139	benzonitrile	ORG	Pseudomonas putida
27817720	biphenyl	ORG	Wautersia oxalatica
953231	catechol	ORG	Ralstonia pickettii
25028858	catechol-ortho derivative	ORG	Corynebacterium efficiens YS-314
25029448	catechol-ortho derivative	ORG	Corynebacterium efficiens YS-314
6707658	catechol-ortho derivative	ORG	Thermobifida fusca
22203519	cresol	ORG	Pseudomonas mendocina
23015058	cyanuric acid	ORG	Magnetospirillum magnetotacticum
18495817	cyclohexanol	ORG	Xanthobacter flavus
567871	dibenzothiophene	ORG	Rhodococcus sp.
21229694	dichloroethane	ORG	Xanthomonas campestris pv. campestris str. ATCC 33913
16762987	DMSO	ORG	Salmonella enterica subsp. enterica serovar Typhi str. CT18
29141870	DMSO	ORG	Salmonella enterica subsp. enterica serovar Typhi Ty2
29144474	DMSO	ORG	Salmonella enterica subsp. enterica serovar Typhi Ty2
3550667	gentisate	ORG	Sphingomonas sp. RW5
4587228	g-hexachlorocyclohexane	ORG	Sphingomonas paucimobilis
17546081	haloacid	ORG	Ralstonia solanacearum GMI1000
27376711	nitrilotriacetate	ORG	Bradyrhizobium japonicum USDA 110
31794257	nitrobenzene	ORG	Mycobacterium bovis AF2122/97
23126227	phenylpropionate	ORG	Nostoc punctiforme
23109223	phenylpropionate	ORG	Azotobacter vinelandii
23017361	phenylpropionate	ORG	Magnetospirillum magnetotacticum
23101892	phenylpropionate	ORG	Azotobacter vinelandii
23062669	phenylpropionate	ORG	Leuconostoc mesenteroides subsp. mesenteroides ATCC 8293

23109194	phenylpropionate	ORG	Azotobacter vinelandii
23107737	phenylpropionate	ORG	Azotobacter vinelandii
23108203	phenylpropionate	ORG	Azotobacter vinelandii
23109691	phenylpropionate	ORG	Azotobacter vinelandii
903977	phthalate	ORG	Burkholderia cepacia
22975371	protocatechuate	ORG	Rhodobacter sphaeroides
14585922	protocatechuate	ORG	Silicibacter pomeroyi
22975204	protocatechuate	ORG	Rhodobacter sphaeroides
7649289	protocatechuate	ORG	Sagittula stellata
19553597	protocatechuate	ORG	Corynebacterium glutamicum ATCC 13032
39934081	protocatechuate	ORG	Rhodopseudomonas palustris CGA009
33333869	pyrene	ORG	Mycobacterium sp. S65
17545808	salicylate	ORG	Ralstonia solanacearum GMI1000
32170713	salicylate	ORG	Streptomyces sp. WA46
38490075	vanillin	ORG	Roseovarius nubinhibens

Rock Sample 142

Genbank ID	Gene short description	Gene category	Organism
16764956	cellulase	CDEG	Salmonella typhimurium LT2
662361	cellulase	CDEG	Pectobacterium carotovorum
12584219	cellulase	CDEG	Volvariella volvacea
520821	cellulase	CDEG	Fusarium oxysporum
21219953	cellulase	CDEG	Streptomyces coelicolor A3(2)
8980306	cellulase	CDEG	Clavibacter michiganensis
23117984	chitinase	CDEG	Desulfitobacterium hafniense
34499695	chitinase	CDEG	Chromobacterium violaceum ATCC 12472
33284730	chitinase	CDEG	Salinivibrio costicola
7209516	chitinase	CDEG	Burkholderia gladioli
21220964	chitinase	CDEG	Streptomyces coelicolor A3(2)
21616730	laccase	CDEG	Trametes sp. C30
1100246	laccase	CDEG	Trametes villosa
4098195	laccase	CDEG	Pleurotus ostreatus (oyster mushroom)
28200469	mannanase	CDEG	Cellvibrio japonicus
40645341	mannanase	CDEG	Paecilomyces lilacinus (nematode egg-parasitic fungus)
34366094	polygalacturonase	CDEG	Aspergillus aculeatus
3982838	polygalacturonase	CDEG	Penicillium griseoroseum

14324459	acsA	CFIX	Staphylococcus aureus subsp. aureus Mu50
25900628	FTHFS	CFIX	Desulfovibrio salexigens
38200102	FTHFS	CFIX	Corynebacterium diphtheriae
37791343	rbcL	CFIX	uncultured proteobacterium
7229168	rbcL	CFIX	uncultured deep-sea autotrophic bacterium TAGI-2
38479625	rbcL	CFIX	Methylocapsa acidiphila
25990790	dsrA	DSR	uncultured bacterium
40253034	dsrA	DSR	uncultured sulfate-reducing bacterium
13898437	dsrA	DSR	uncultured phenanthrene mineralizing bacterium
14276799	dsrA	DSR	Desulfotomaculum geothermicum
13898429	dsrA	DSR	uncultured phenanthrene mineralizing bacterium
34017156	dsrA	DSR	uncultured bacterium
34017094	dsrA	DSR	uncultured bacterium
902747	dsrA	DSR	Desulfovibrio vulgaris
2576394	dsrB	DSR	Allochromatium vinosum
FW010103B	dsrB	DSR	lab clone
13249523	dsrB	DSR	uncultured sulfate-reducing bacterium
3095051	arsenic	MET	Pseudomonas aeruginosa
22958951	arsenic	MET	Rhodobacter sphaeroides
23473907	arsenic	MET	Desulfovibrio desulfuricans G20
23054509	arsenic	MET	Leuconostoc mesenteroides subsp. mesenteroides ATCC 8293
23102528	arsenic	MET	Azotobacter vinelandii
27314796	arsenic	MET	Staphylococcus epidermidis ATCC 12228
17530581	arsenic	MET	IncN plasmid R46
29840655	cadmium	MET	Chlamydophila caviae GPIC
21244878	cadmium	MET	Xanthomonas axonopodis pv. citri str. 306
13898625	cadmium	MET	Pseudomonas putida
34496726	chromium	MET	Chromobacterium violaceum ATCC 12472
23012809	chromium	MET	Magnetospirillum magnetotacticum
22986503	chromium	MET	Rhodobacter sphaeroides
22975267	chromium	MET	Rhodobacter sphaeroides
22981959	chromium	MET	Rhodobacter sphaeroides
32039492	chromium	MET	Pseudomonas aeruginosa UCBPP-PA14
17548712	cobalt,zinc,cadmium	MET	Ralstonia solanacearum GMI1000
14331038	copper	MET	Pseudomonas fluorescens
10047065	copper	MET	Wautersia metallidurans

10047068	copper	MET	Wautersia metallidurans
10047069	copper	MET	Wautersia metallidurans
17548879	copper	MET	Ralstonia solanacearum GMI1000
17937687	copper	MET	Agrobacterium tumefaciens str. C58
21225358	copper	MET	Streptomyces coelicolor A3(2)
39996164	cytochrome	MET	Geobacter sulfurreducens PCA
39934990	cytochrome	MET	Rhodopseudomonas palustris CGA009
24372200	cytochrome	MET	Shewanella oneidensis MR-1
39933281	cytochrome	MET	Rhodopseudomonas palustris CGA009
39996386	cytochrome	MET	Geobacter sulfurreducens PCA
20258071	cytochrome	MET	Rhodopseudomonas palustris
39998003	cytochrome	MET	Geobacter sulfurreducens PCA
18076024	mercury	MET	Pseudomonas sp.
24411186	mercury	MET	Acinetobacter sp. ED23-35
23821233	mercury	MET	Pseudomonas putida
24372984	mercury	MET	Shewanella oneidensis MR-1
18076026	mercury	MET	Pseudomonas sp.
6689528	mercury	MET	Xanthomonas campestris
4572426	mercury	MET	Sphingomonas paucimobilis
15920013	mercury	MET	Plasmid pSB102
17549265	nickel	MET	Ralstonia solanacearum GMI1000
28870264	nickel	MET	Pseudomonas syringae pv. tomato str. DC3000
17225098	nickel,cobalt	MET	Hafnia alvei
19554054	tellurium	MET	Corynebacterium glutamicum ATCC 13032
34498563	tellurium	MET	Chromobacterium violaceum ATCC 12472
32474566	tellurium	MET	Pirellula sp. 1
22988087	tellurium	MET	Rhodobacter sphaeroides
22979636	tellurium	MET	Rhodobacter sphaeroides
23000799	tellurium	MET	Xylella fastidiosa Ann-1
28868161	tellurium	MET	Pseudomonas syringae pv. tomato str. DC3000
21222139	tellurium	MET	Streptomyces coelicolor A3(2)
38016653	tellurium	MET	Klebsiella pneumoniae
23057746	tellurium	MET	Leuconostoc mesenteroides subsp. mesenteroides ATCC 8293
4126672	zinc	MET	Staphylococcus aureus
1749680	zinc,cadmium	MET	Schizosaccharomyces pombe (fission yeast)
126867	mcr	methane_gen	Methanothermobacter marburgensis str. Marburg

34305102	mcrA	methane_gen	uncultured archaeon
6002402	mmo	methane_ox	Methylomonas sp. KSPIII
2098700	mmo	methane_ox	Methylocystis sp. M
6424923	pmoA	methane_ox	uncultured eubacterium pAMC512
7188931	pmoA	methane_ox	Methylosinus trichosporium
4105847	pmoA	methane_ox	thermophilic methanotroph HB
10863141	nifH	NFIX	marine stromatolite eubacterium HB(0697) A100
20278769	nifH	NFIX	uncultured nitrogen-fixing bacterium
20090488	nifH	NFIX	Methanosarcina acetivorans C2A
20804123	nifH	NFIX	Mesorhizobium loti
6523533	nifH	NFIX	uncultured microorganism SN-12
23058236	0	NIT	Leuconostoc mesenteroides subsp. mesenteroides ATCC 8293
15616504	gdh	NIT	Bacillus halodurans C-125
5881154	urease	NIT	Sporosarcina pasteurii
15966216	urease	NIT	Sinorhizobium meliloti 1021
13474124	urease	NIT	Mesorhizobium loti MAFF303099
32033449	urease	NIT	Actinobacillus pleuropneumoniae serovar 1 str. 4074
23011399	urease	NIT	Magnetospirillum magnetotacticum
14024887	urease	NIT	Mesorhizobium loti
19070378	urease	NIT	Rhizobium leguminosarum bv. viciae
38605266	urease	NIT	Bradyrhizobium japonicum
21219745	urease	NIT	Streptomyces coelicolor A3(2)
13474125	urease	NIT	Mesorhizobium loti MAFF303099
37526091	urease	NIT	Photorhabdus luminescens subsp. laumondii TTO1
29610775	urease	NIT	Streptomyces avermitilis MA-4680
38605398	urease	NIT	Ralstonia solanacearum
14024877	urease	NIT	Mesorhizobium loti
23005331	urease	NIT	Magnetospirillum magnetotacticum
17982573	urease	NIT	Brucella melitensis 16M
37731823	urease	NIT	Nitrosomonas cryotolerans
17740883	urease	NIT	Agrobacterium tumefaciens str. C58
34496646	urease	NIT	Chromobacterium violaceum ATCC 12472
2440024	urease	NIT	Wautersia eutropha
12007388	narB	NRED	Synechococcus sp. WH 7803
14348604	narG	NRED	Paracoccus pantotrophus
38427022	narG	NRED	uncultured bacterium

26278684	narG	NRED	uncultured bacterium
26278882	narG	NRED	uncultured bacterium
29652432	narG	NRED	uncultured bacterium
26278876	narG	NRED	uncultured bacterium
24983618	nasA	NRED	Pseudomonas putida KT2440
16120504	nirB	NRED	Yersinia pestis CO92
12597209	nirK	NRED	Alcaligenes sp. STC1
27125563	nirK	NRED	uncultured bacterium
3758830	nirK	NRED	Hyphomicrobium zavarzinii
37999162	nirS	NRED	uncultured bacterium
24421427	nirS	NRED	uncultured organism
29466092	norB	NRED	uncultured bacterium
3057083	nosZ	NRED	Paracoccus pantotrophus
5688873	2,4,5-T	ORG	Arthrobacter sp.
39935218	2,4,5-T	ORG	Rhodopseudomonas palustris CGA009
9820076	3-chlorobenzoate	ORG	Comamonas testosteroni
3063589	acetylene	ORG	Synechococcus sp. PCC 7002
24575091	acetylene	ORG	Streptomyces globisporus
40890131	acrylonitrile	ORG	uncultured organism
40890225	acrylonitrile	ORG	uncultured organism
40890327	acrylonitrile	ORG	uncultured organism
40890319	acrylonitrile	ORG	uncultured organism
40890261	acrylonitrile	ORG	uncultured organism
40890297	acrylonitrile	ORG	uncultured organism
27657375	acrylonitrile	ORG	uncultured bacterium BD1
18976382	aminocyclopropane	ORG	Pyrococcus furiosus DSM 3638
1841362	aniline	ORG	Pseudomonas putida
7542440	benzoate-aerobic	ORG	Pseudomonas putida
14289342	benzoate-aerobic	ORG	Rhodococcus sp. 19070
25990733	benzoate-aerobic	ORG	Streptomyces setonii
14289340	benzoate-aerobic	ORG	Rhodococcus sp. 19070
31407696	benzoate-aerobic	ORG	Acinetobacter calcoaceticus
7542439	benzoate-aerobic	ORG	Pseudomonas putida
18369658	benzoate-anaerobic	ORG	Azoarcus evansii
15836922	benzoate-anaerobic	ORG	Xylella fastidiosa 9a5c
13473296	benzoate-anaerobic	ORG	Mesorhizobium loti MAFF303099

33603723	benzoate-anaerobic	ORG	Bordetella bronchiseptica RB50
33595881	benzoate-anaerobic	ORG	Bordetella parapertussis 12822
218366	benzoate-anaerobic	ORG	Candida tropicalis
2190579	benzoate-anaerobic	ORG	Rhodopseudomonas palustris CGA009
33597722	benzoate-anaerobic	ORG	Bordetella parapertussis 12822
1877504	benzonitrile	ORG	Pseudomonas putida
391834	biphenyl	ORG	Pseudomonas sp.
28193603	biphenyl	ORG	Ralstonia sp. SBUG 290
27228520	carbazole	ORG	Pseudomonas resinovorans
3243172	carbazole	ORG	Sphingomonas sp. CB3
6048992	catechol	ORG	Burkholderia sp. RP007
3402316	catechol	ORG	unidentified bacterium
3402328	catechol	ORG	unidentified bacterium
4579698	catechol	ORG	Frateuria sp. ANA-18
23094407	catechol-ortho derivative	ORG	Rhodococcus opacus
3025224	catechol-ortho derivative	ORG	Methylobacterium extorquens
17936409	catechol-ortho derivative	ORG	Agrobacterium tumefaciens str. C58
23124219	catechol-ortho derivative	ORG	Nostoc punctiforme
24985882	catechol-ortho derivative	ORG	Pseudomonas putida KT2440
27228503	catechol-ortho derivative	ORG	Pseudomonas resinovorans
38638066	catechol-ortho derivative	ORG	Wautersia eutropha
5305775	catechol-ortho derivative	ORG	Mycobacterium smegmatis
12003192	chlorophenol	ORG	Desulfitobacterium chlororespirans
23060646	cyanuric acid	ORG	Leuconostoc mesenteroides subsp. mesenteroides ATCC 8293
22995618	cyanuric acid	ORG	Xylella fastidiosa Dixon
23102161	cyanuric acid	ORG	Azotobacter vinelandii
13488245	dibenzothiophene	ORG	Mesorhizobium loti MAFF303099
17937156	dibenzothiophene	ORG	Agrobacterium tumefaciens str. C58
40316488	dibenzothiophene	ORG	Bacillus subtilis
15625037	dibenzothiophene	ORG	Rhodovulum sulfidophilum
26986209	dibenzothiophene	ORG	Paracoccus versutus
16759573	DMSO	ORG	Salmonella enterica subsp. enterica serovar Typhi str. CT18
29141870	DMSO	ORG	Salmonella enterica subsp. enterica serovar Typhi Ty2
29142390	DMSO	ORG	Salmonella enterica subsp. enterica serovar Typhi Ty2
30268636	fluorene	ORG	Terrabacter sp. DBF63
3550667	gentisate	ORG	Sphingomonas sp. RW5

32037450	gentisate	ORG	Pseudomonas aeruginosa UCBPP-PA14
4521186	g-hexachlorocyclohexane	ORG	Sphingomonas paucimobilis
27375220	homogentisate-derivative	ORG	Bradyrhizobium japonicum USDA 110
17549466	hydroxyacetophenone	ORG	Ralstonia solanacearum GMI1000
15420769	MTBE	ORG	Rhodococcus sp. Q15
17544992	naphthalene	ORG	Ralstonia solanacearum GMI1000
9957198	nitrilotriacetate	ORG	EDTA-degrading bacterium BNC1
16804381	nitrilotriacetate	ORG	Listeria monocytogenes EGD-e
17937158	nitrilotriacetate	ORG	Agrobacterium tumefaciens str. C58
6014660	nitrobenzoate	ORG	Ralstonia pickettii
3115396	pentachlorophenol	ORG	Sphingomonas sp. UG30
16604298	pentachlorophenol	ORG	Sphingomonas sp. UG30
3820513	phenanthrene	ORG	Burkholderia sp. RP007
37519361	phenanthrene	ORG	Pseudomonas fluorescens
7619821	phenanthrene	ORG	Nocardioides sp. KP7
31407691	phenol-aerobic	ORG	Acinetobacter calcoaceticus
5851788	phenol-aerobic	ORG	Ralstonia sp. KN1
14276040	phenol-aerobic	ORG	uncultured microorganism PCRHD10
32967108	phenol-aerobic	ORG	Pseudomonas sp. KL28
17826940	phenol-aerobic	ORG	Burkholderia kururiensis
23059516	phenylpropionate	ORG	Leuconostoc mesenteroides subsp. mesenteroides ATCC 8293
23109223	phenylpropionate	ORG	Azotobacter vinelandii
23468765	phenylpropionate	ORG	Pseudomonas syringae pv. syringae B728a
23101892	phenylpropionate	ORG	Azotobacter vinelandii
23106474	phenylpropionate	ORG	Azotobacter vinelandii
23109194	phenylpropionate	ORG	Azotobacter vinelandii
22983104	phenylpropionate	ORG	Rhodobacter sphaeroides
30063928	phenylpropionate	ORG	Shigella flexneri 2a str. 2457T
22987953	phenylpropionate	ORG	Rhodobacter sphaeroides
903977	phthalate	ORG	Burkholderia cepacia
4128218	phthalate	ORG	Burkholderia cepacia
29828245	protocatechuate	ORG	Streptomyces avermitilis MA-4680
14585922	protocatechuate	ORG	Silicibacter pomeroyi
22975204	protocatechuate	ORG	Rhodobacter sphaeroides
17546160	protocatechuate	ORG	Ralstonia solanacearum GMI1000
14585964	protocatechuate	ORG	marine bacterium SE96

21230278	protocatechuate	ORG	Xanthomonas campestris pv. campestris str. ATCC 33913
22978566	protocatechuate	ORG	Rhodobacter sphaeroides
33333869	pyrene	ORG	Mycobacterium sp. S65
17545808	salicylate	ORG	Ralstonia solanacearum GMI1000
10957461	salicylate	ORG	Deinococcus radiodurans R1
39935184	thiocyanate	ORG	Rhodopseudomonas palustris CGA009
20137979	thiocyanate	ORG	Pseudomonas aeruginosa
2498273	thiocyanate	ORG	Synechocystis sp. PCC 6803
22002034	thiocyanate	ORG	Oligotropha carboxidovorans
151593	toluene-aerobic	ORG	Pseudomonas mendocina
22203518	toluene-aerobic	ORG	Pseudomonas mendocina
4929532	toluene-aerobic	ORG	Acinetobacter sp. ADP1
17221316	toluene-anaerobic	ORG	Thauera aromatica
17548444	vanillin	ORG	Ralstonia solanacearum GMI1000
17988985	vanillin	ORG	Brucella melitensis 16M
40788119	vanillin	ORG	Pseudomonas fluorescens
22127923	xylene	ORG	Yersinia pestis KIM
