# Supplementary material for: First Investigation of the Microbiology of the Deepest Layer of Ocean Crust
Source: PLoS One. 2010 Nov 5;5(11):e15399. doi: 10.1371/journal.pone.0015399 (PMC2974637; doi:10.1371/journal.pone.0015399)
Supplement: Methods S1 [file pone.0015399.s003.doc]

**Supplementary Methods**

T-RFLP peaks greater than 50 bp was standardized using the variable percentage

threshold method described by Osborne et al. (2006). Standardized data was transformed using the power transformation function in PC-ORD (McCune and Grace, 2002). Shannon diversity indices were calculated using PC-ORD.

**References**

McCune, B., and Grace, J.B. (2002) *Analysis of Ecological Communities*: MJM Software.

Osborne, C.A., Rees, G.N., Bernstein, Y., and Janssen, P.H. (2006) New Threshold and

Confidence Estimates for Terminal Restriction Fragment Length Polymorphism Analysis

of Complex Bacterial Communities. *Appl. Environ. Microbiol.* 72: 1270-1278.
